# Metabolic Behaviors of Aconitum Alkaloids in Different Concentrations of *Aconiti Lateralis Radix Praeparata* and Effects of Aconitine in Healthy Human and Long QT Syndrome Cardiomyocytes

**DOI:** 10.3390/molecules27134055

**Published:** 2022-06-23

**Authors:** Liang Yang, Guanghui Xie, Yuguang Wang, Jian Li, Bin Zheng, Jinmiao Zhu, Xinsong Yuan, Qian Hong, Zengchun Ma, Yue Gao

**Affiliations:** 1 School of Chemistry and Chemical Engineering, Hefei Normal University, Hefei 230601, China; yangliang@hfnu.edu.cn (L.Y.); zhengbin@hfnu.edu.cn (B.Z.); zhujinmiao@hnu.edu.com (J.Z.); yuanxs@zju.edu.cn (X.Y.); 2 Beijing Institution of Radiation Medicine, Beijing 100850, China; xgh181075@163.com (G.X.); wangyg@bmi.ac.cn (Y.W.); lijianky@163.com (J.L.); 3 Huaihai Hospital, Xuzhou Medical University/PLA 71st Group Military Hospital, Xuzhou 221004, China; hongqian177@126.com

**Keywords:** *Fu Zi*, pharmacokinetic, UPLC-MS/MS, Long QT syndrome, aconitine, hiPSC-CMs

## Abstract

Aconiti Lateralis Radix Praeparata (*Fu Zi*) is the processed lateral root of *Aconitum carmichaelii* Debx, which is widely used in emergency clinics. Poisoning incidents and adverse reactions occur with the improper intake of *Fu Zi.* Metabolic characteristics of aconitum alkaloids of *Fu Zi* may vary, and the effects of *Fu Zi* in healthy and Long QT syndrome (LQTS) patients is unknown. In this experiment, 24 Sprague Dawley rats were randomly divided into three groups: 2.0, 1.0, and 0.5 g/kg dose groups, and blood samples were collected after the oral administration of *Fu Zi* extract. We used an ultra-high performance liquid chromatography-tandem mass spectrometry system to detect the concentrations of six aconitum alkaloids. Cell toxicity, calcium imaging, and patch-clamp recordings of human induced pluripotent stem cells-cardiomyocytes (hiPSC-CMs) of aconitine in healthy and LQTS were observed. We found that the AUC_(0–48h)_, C_max_, and t_1/2_ of the six compounds increased with the multiplicative dosages; those in the high group were significantly higher than those in the low group. Aconitine concentration-dependently decreased the amplitude, which has no significant effect on the cell index of normal hiPSC-CMs. Aconitine at 5.0 μM decreased the cell index between 5–30 min for LQTS hiPSC-CMs. Meanwhile, aconitine significantly increased the frequency of calcium transients in LQTS at 5 μM. Aconitine significantly shortened the action potential duration of human cardiomyocytes in both normal and LQTS groups. These results show metabolic behaviors of aconitum alkaloids in different concentrations of *Fu Zi* and effects of aconitine in healthy and LQTS patients.

## 1. Introduction

**Figure 1:**
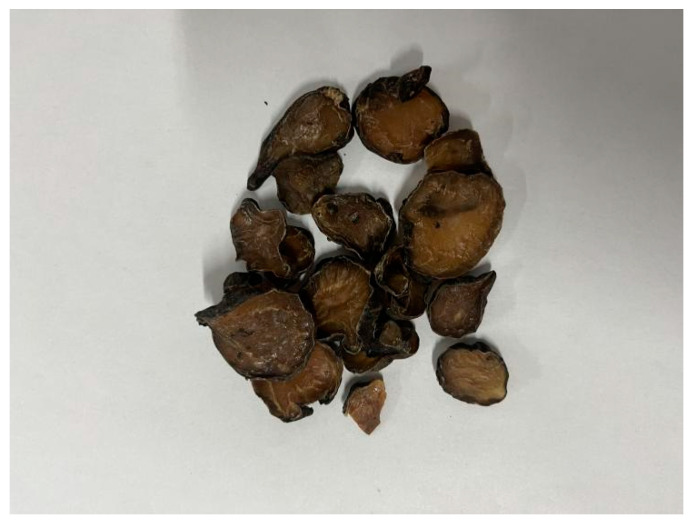
*Fu Zi* specimens (processed lateral root of *Aconitum carmichaelii* Debx).

**Figure 2:**
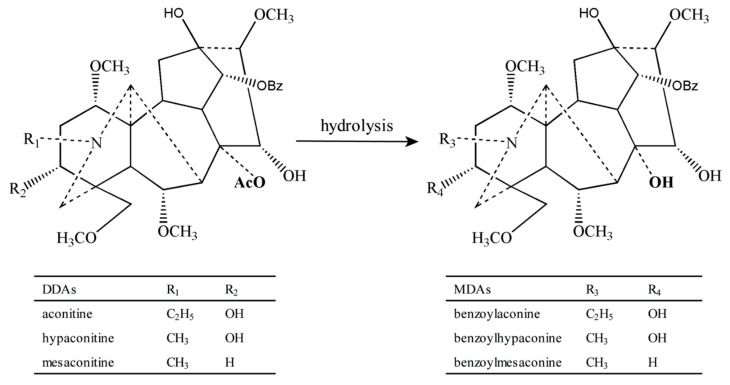
Chemical structures of the six aconitum alkaloids, and the reaction from DDAs to MDAs.

Traditional Chinese Medicine (TCM) is now practiced in many countries, such as the United States, Canada, and Europe [1], and it has been proposed to possess great potential to improve people’s health and wellness [2,3]. *Aconitum* carmichaelii Debx of the *genus Aconitum L.* (Ranunculaceae) is an example of TCM that has been used for 2000 years. Aconiti Lateralis Radix Praeparata (*Fu Zi*) is the processed lateral root of *Aconitum carmichaelii* Debx (Figure 1) [4,5,6,7]. The active aconitum alkaloids in *Fu Zi* encompass diester-diterpene alkaloids (DDAs): aconitine (AC), hypaconitine (HA), mesaconitine (MA), and monoester-diterpene alkaloids (MDAs), which include benzoylaconine (BAC), benzoylhypaconine (BHA), and benzoylmesaconine (BMA) [8,9]. DDAs can be converted into MDAs through hydrolysis [10,11,12] (Figure 2). Numerous studies have reported that poisoning incidents and adverse reactions occur with the improper intake of *Fu Zi*. The typical poisoning symptoms of *Fu Zi* include: arrhythmia, vomiting, nausea, hypotension, dizziness, and coma. The clinical application of *Fu Zi* has obvious effects of low-dose treatment and high-dose toxicity, namely neurotoxicity and cardiotoxicity, which limit the safe application of *Fu Zi* in clinical therapeutics [13,14,15,16,17]. Thus, the dosage of *Fu Zi* is strictly limited in the Chinese Pharmacopoeia (2020) to 3–15 g/person/day. The pharmacokinetics of *Fu Zi* extracts have been extensively studied and metabolic kinetic evaluation of AC or multiple alkaloids in a single dose is known [18,19,20,21]; however, the effects of multiple doses on the pharmacokinetics of six aconitum alkaloids of *Fu Zi* extracts have not yet been reported. Considering that hundreds of Chinese herbal medicines have numerous chemical molecules, quality markers were proposed for quality assessment. The contents of DDAs and MDAs in *Fu Zi* are limited in the Chinese Pharmacopoeia 2020 edition, and the upper limit of DDAs (less than 0.020%) and lower limit of MDAs (more than 0.010%) are stipulated, so that these six aconitum alkaloids could serve as quality marker compounds for the chemical evaluation of *Fu Zi*. Meanwhile, many clinical reports have shown that the same dose of AC has different effects on patients with varied physical conditions. The mechanism is controversial. In our preliminary experiments, we found that the toxic effects of AC on normal cells showed strong tolerance in doxorubicin-induced cardiac injury; however, models of cardiac injury of rat cardiomyocytes, human cardiomyocytes, or entire animals have great limitations [22]. Human induced pluripotent stem cells (hiPSCs) make it possible to rapidly detect the toxicity of compounds in vitro. The cardiomyocytes of different disease models were established by reprogramming epithelial cells obtained from patients with cardiac diseases. Compared to primary cardiomyocytes constructed in vitro, hiPSCs preserve the characteristics of the patient’s disease, which has structural and functional similarities with native human cardiomyocytes. Cardiac disease models on hiPSCs is highly consistent with the pathogenesis of clinical patients, and it has been widely used to evaluate the early screening and mechanism of toxic drugs in recent years. Long QT syndrome (LQTS) refers to a group of syndromes that lead to malignant ventricular arrhythmia, especially tip torsional ventricular tachycardia and sudden death due to prolonged QT interval and abnormal T wave in electrocardiograms; these syndromes can be divided into congenital long QT syndrome (cLQTS) and acquired LQTS [23]. The most common clinical cases are LQT1, LQT2, and LQT3, which account for about 75% of LQTS patients [24]. Researchers have found that the same dose of aconite showed a toxic effect on normal subjects and a therapeutic effect on patients with arrhythmia, and this toxic effect manifests in a variety of forms, which suggests a potential arrhythmic effect, accounting for acquired LQTS [25].

Thus, the present study aims to establish an ultra-high performance liquid chromatography-tandem mass spectrometry (UPLC-MS/MS) method for the simultaneous determination of six aconitum alkaloids in rat plasma, to obtain valuable pharmacokinetic data, and to compare different effects of AC on normal and LQTS hiPSCs.

## 2. Results and Discussion

### 2.1. Method Validation

#### 2.1.1. Selectivity

**Figure 3:**
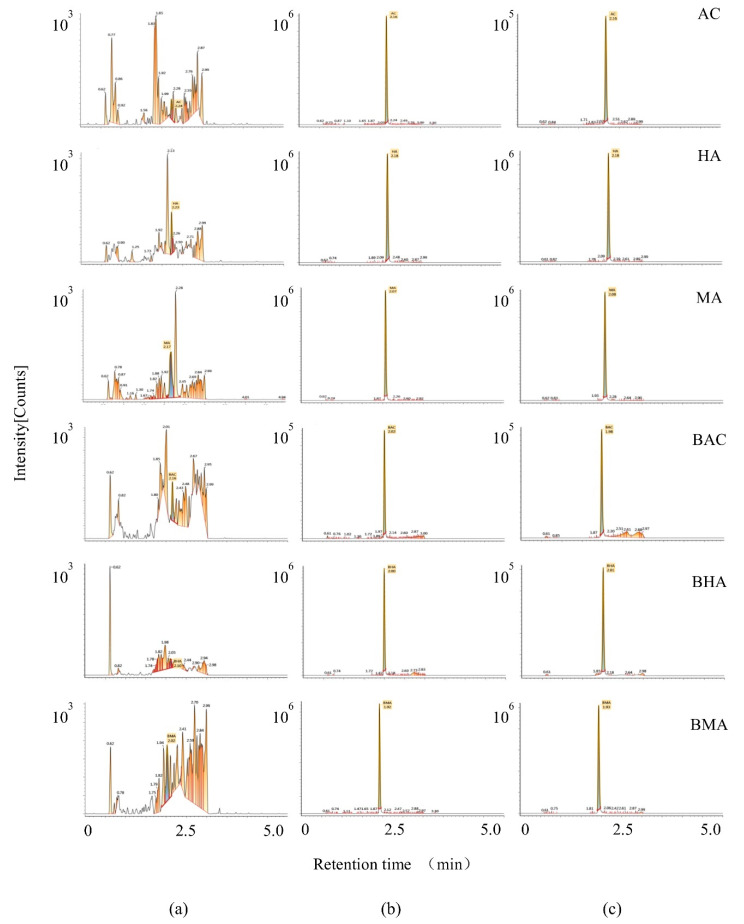
Multiple reaction monitoring (MRM) chromatograms of AC, HA, MA, BAC, BHA, BMA, (**a**) Blank plasma; (**b**) Blank plasma spiked with the six compounds; (**c**) A rat plasma sample after oral administration of *Fu Zi* extracts (1.0 g/kg).

The selectivity of the method was determined by comparing the chromatograms of blank, spiked, and experiment plasma samples after the dosing of *Fu Zi* extracts. The retention time of AC, HA, MA, BAC, BHA, and BMA was 2.16, 2.18, 2.08, 2.03, 2.01, and 1.93 min, respectively, which demonstrated that there was no endogenous interference at the same retention times (Figure 3).

#### 2.1.2. Sensitivity and Linearity

The sensitivity was defined by the six spiked samples at a lower limit of quantitation (LLOQ) concentration. The signal-to-noise ratio of limit of detection (LOD) was above three. The linearity was accessed by peak areas of seven points on the optimal calibration curve and established by the weighted least squares linear regression. The calibration equations, linear ranges, coefficients, LLOQ, and LOD were measured and calculated (Table 1).

#### 2.1.3. Accuracy and Precision

The data for accuracy and precision were measured and calculated. The inter-day accuracy [Relative error, RE (%)] of MA at LLOQ was −15.7%. The other accuracy of both inter-day and intra-day for six compounds were within ± 14.7%. The precision [Relative standard deviation, RSD (%)] of BAC at LLOQ was 18.8% and the other compounds ranged from 4.7–13.6% (Table 2). The data indicated that the methods had an optimal accuracy and reproducibility of the method.

#### 2.1.4. Stability

The RE of the six compounds at different conditions varied from 12.2–11.4% and the RSDs ranged from 2.9–13.1% (Table 3). The samples were stable at different storage conditions, such as at room temperature for 4 h, at the autosampler for 10 h, at −20 °C for 1 week, and after three freeze-thaw cycles.

#### 2.1.5. Extraction Recovery and Matrix Effect

The extraction recovery of the six compounds ranged from 67.7 ± 4.0 to 101.1 ± 5.5%, and the matrix effects varied from 67.6 ± 8.8 to 100.3 ± 6.9%. In addition, the RSDs ranged from 4.5% to 9.4% for extraction recovery and 6.1% to 14.0% for matrix effect, which fulfilled the criteria (Table 4). These results demonstrated that the approach of protein precipitation was acceptable, and the peaks of the endogenous matrix had no effect on the quantification of the six compounds.

### 2.2. Pharmacokinetic Study

**Figure 4:**
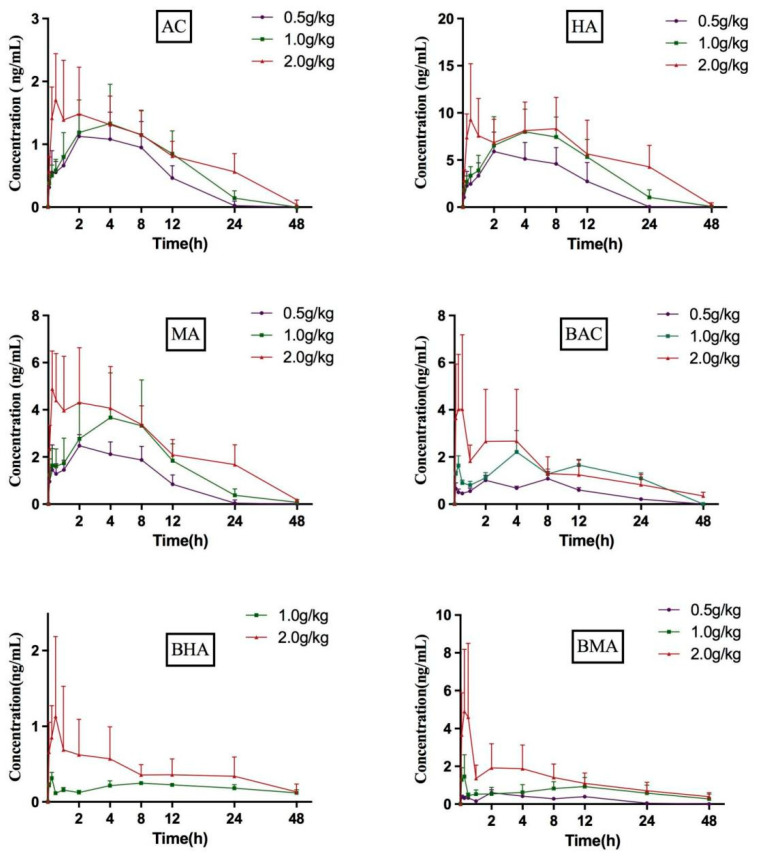
Plasma concentration–time profiles of the six compounds in SD rat plasma at different oral dose of 0.5 g/kg, 1.0 g/kg and 2.0 g/kg of *Fu Zi* extracts (*n* = 8), note: concentration of BHA at 0.5 g/kg was Below the Quantization Limit.

When applied to the pharmacokinetic assessment, the validated method was suitable for the quantification of the six compounds in SD rat plasma at different oral doses of 2.0 g/kg, 1.0 g/kg, and 0.5 g/kg of *Fu Zi* extracts. The mean plasma concentration–time profiles of the six compounds were tested (Figure 4), and the main pharmacokinetic parameters were calculated (Table 5). Briefly, the AUC_(0–48h)_, C_max_, and t_1/2_ of the six compounds increased with the multiplicative dosages; those in the high group were significantly higher than those in the low group. The plasma concentrations of BHA in the low group were below LLOQ at most of the time points.

### 2.3. Cell Toxicity of AC on Normal and LQTS hiPSC-CMs

AC, MA, and HA have a similar structure and function, and because most researchers of the prior *Fu Zi* toxicity-effect studies selected AC as the representative component, cell toxicity of AC on normal and LQTS hiPSC-CMs. were also examined.

**Figure 5:**
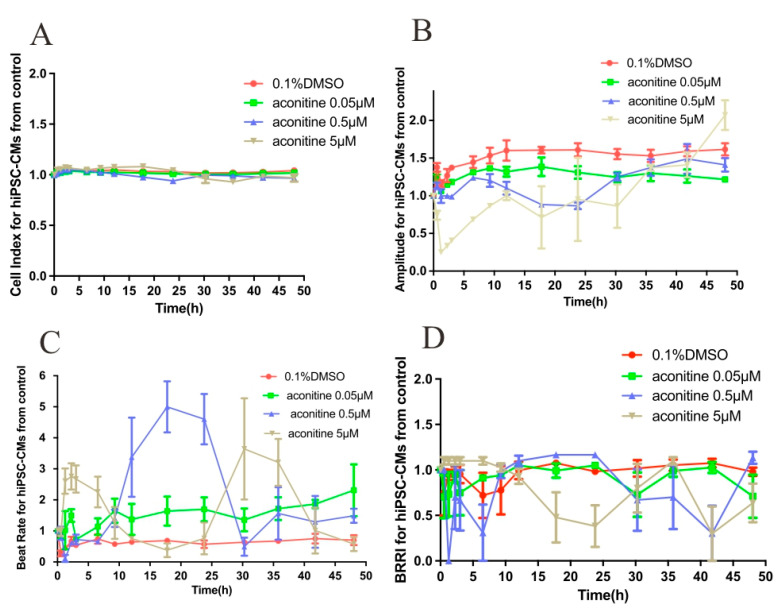

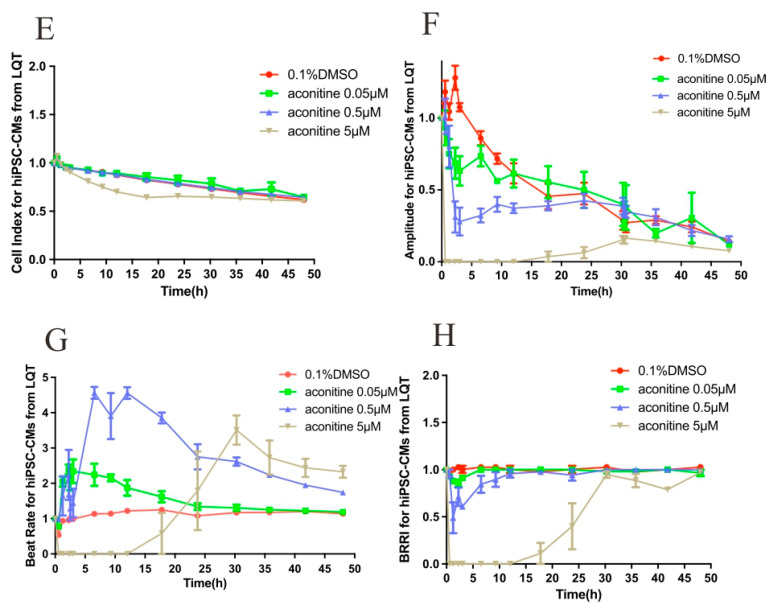
High-throughput analysis of AC toxicity in hiPSC-CMs from control or LQT syndrome. (**A**) Cell index of normal hiPSC-CMs exposed to AC; (**B**) Cell beat amplitude of normal hiPSC-CMs exposed to AC; (**C**) Cell beat frequency of normal hiPSC-CMs exposed to AC; (**D**) Cell beat rate of normal hiPSC-CMs exposed to AC; (**E**) Cell index of LQT syndrome hiPSC-CMs exposed to AC; (**F**) Cell beat amplitude of LQT syndrome hiPSC-CMs exposed to AC; (**G**) Cell beat frequency of LQT syndrome hiPSC-CMs exposed to AC; (**H**) Cell beat rate of LQT syndrome hiPSC-CMs exposed to AC.

None of the three concentrations (0.05, 0.5, and 5 μM) AC caused cardiomyocyte death during the 48 h observation period (Figure 5A,E). The contraction frequency was slightly higher and the contraction force was slightly lower than the control group (Figure 5B,D,F,H). The concentration of 0.5 μM increased cell frequency and decreased cell contractility (Figure 5B,C,F,G).

Cell contractility was significantly reduced at 5 μM concentration and was very weak within 6 h after treatment. Cell beat rate of normal hiPSC-CMs exposed to AC (0.5 μM) was faster after 12 h, while cell beat frequency of LQT syndrome hiPSC-CMs (AC 0.5 μM) continued to 6 h.

Effects of AC on LQT myocardial cells. None of the three concentrations caused significant myocardial cell death during the 48 h observation period. The concentration of 0.05 μM had a significant effect on the cells, which showed higher contraction frequency and lower contraction force than the control group. The cell frequency and contractility were significantly increased at the 0.5 μM concentration. The cell contractility decreased to zero with the 5 μM concentration, and it was very weak within 30 h after treatment with AC.

### 2.4. Effects of AC on APD in Normal and LQT Syndrome hiPSC-CMs

**Figure 6:**
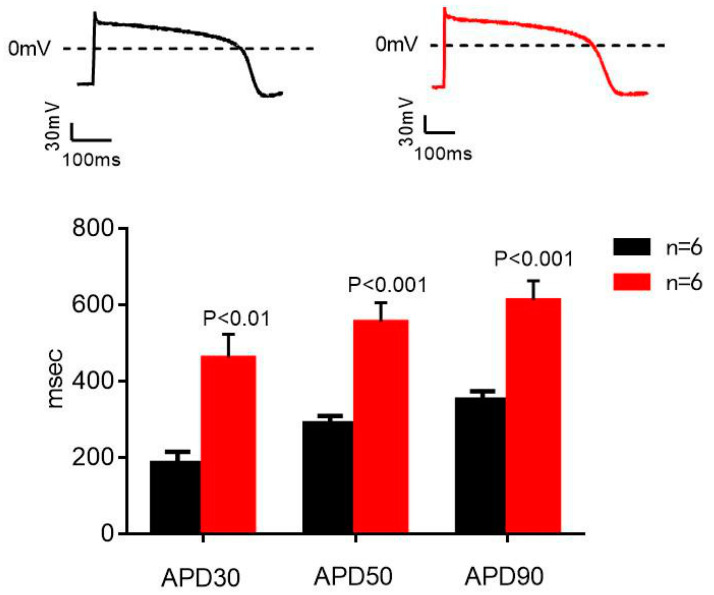
Whole-cell current-clamp recording from hiPSC-CMs. Panel A shows the results of representative AP wave forms recorded in a control and a Long-QT Syndrome (LQTS) hiPSC-CMs. Notice the marked APD prolongation in LQTS hiPSC-CMs. The bar graphs represent the averaged action potential duration at 30% repolarization (APD30) at 50% repolarization (APD50) and at 90% repolarization (APD90) for control and LQTS hiPSC-CMs at a 1-Hz stimulation rate. *p* values are for the comparison between control and LQTS hiPSC-CMs, with the use of independent samples *t*-test. Data are means ± SEM for 6 cells in each group (the control subjects and LQTS subjects). (Black: normal; Red: LQTS).

**Figure 7:**
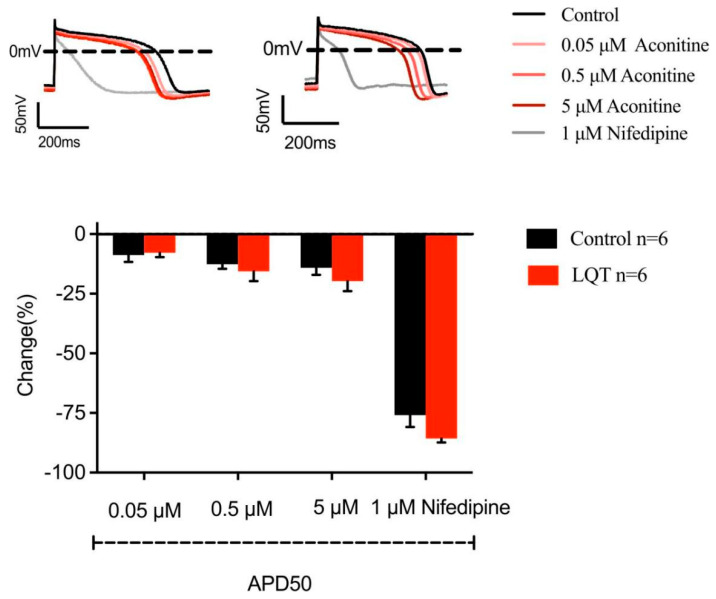
Effects of AC on action potentials of hiPSC-CMs. Panel A shows an overlay of single action potentials from a representative cell from a control hiPSC-CMs and from a long-QT syndrome (LQTS) hiPSC-CMs at a 1 Hz stimulation rate before (basal) and after application of 0.05 μM–5μM AC. The bar graph represents the percent change induced by 0.05 μM–5μM AC and 1 μM nifedipine on action potential duration (APD) at 50% repolarization (APD50). Data are means ± SEM for 6 cells in each group (the control subjects and LQTS subjects) (Black: normal; Red: LQTS).

Figure 6 shows the whole-cell current-clamp recording from hiPSC-CMs and the effects of AC on action potentials of hiPSC-CMs. Panel A shows an overlay of single action potentials from a representative cell from a control hiPSC-CMs and from a long-QT syndrome (LQTS) hiPSC-CMs. It implied that AC (0.05, 0.5, and 5 μM) concentration-dependently shortened APD50 at normal and LQTS hiPSC-CMs. The effects of AC resembled that of nifedipine, a specific LTCC blocker (Figure 7).

### 2.5. Effects of AC on Calcium Transients in Normal and LQT Syndrome hiPSC-CMs

**Figure 8:**
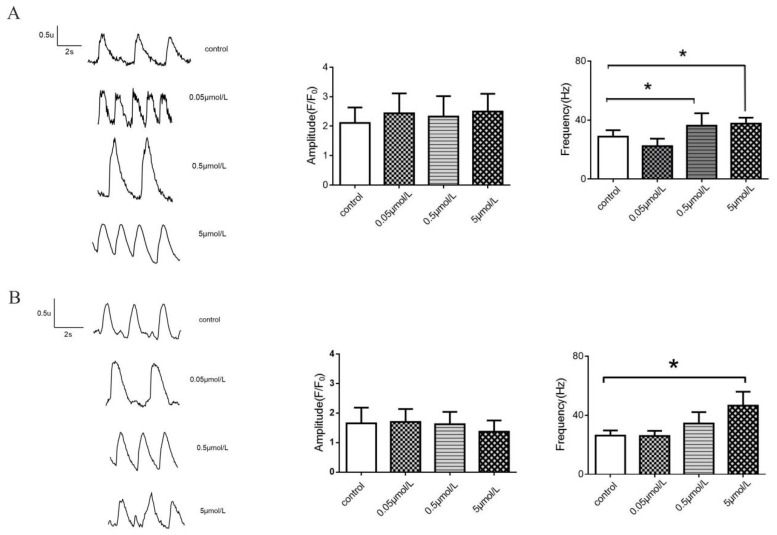
Effects of AC on calcium transients in normal and LQT syndrome hiPSC-CMs. (**A**) normal hiPSC-CMs; (**B**) LQT syndrome hiPSC-CMs. * Compared with the control group, *p* < 0.05.

Compared with the control group, the detected AC had no significant effect on the amplitude and frequency of calcium transient in normal hiPSC-CMs, but it could significantly increase the frequency of calcium transient in cardiomyocytes of LQTS at 5 μM (Figure 8).

### 2.6. Effects of AC on L-Type Calcium Current in Normal and LQTS hiPSC-CMs

**Figure 9:**
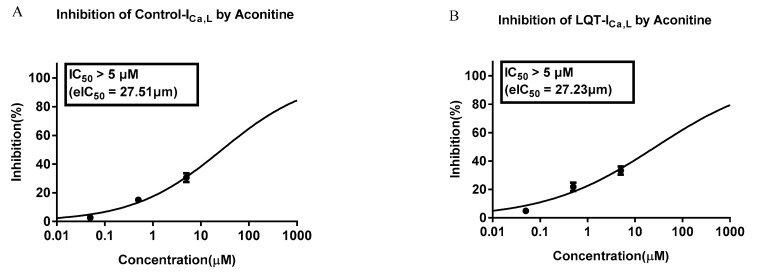
The inhibitory curve of AC at multiple concentrations on L-type calcium current. (**A**) normal hiPSC-CMs; (**B**) LQT syndrome hiPSC-CMs.

The peak value of L-type calcium current of human cardiomyocytes in normal and LQT groups was significantly decreased by 0.05–5 μM AC, with IC_50_ > 5 μM with no concentration dependence. A stronger I_Ca,L_ blocking was noted (Figure 9). The effects of AC on I_Ca,L_ (L-type calcium channel) currents in normal and LQTS hiPSC-CMs were validated in the heterologous expression systems. Using a HEKA EPC-10 patch-clamp system, the effects of AC (0.05–5μM) on I_Ca,L_ were measured in normal and LQTS hiPSC-CMs. AC showed a mild blockage with IC_50_ of 27.51 μM and 27.23 μM for normal and LQTS hiPSC-CMs, respectively.

## 3. Discussion

Considering the complex components and the different doses of *Fu Zi* in prescriptions, multiple components must be analyzed to ensure that *Fu Zi* is used safely in therapeutic treatments. A rapid and accurate method for the determination of three types of DDAs (AC, HA, and MA) was established previously [26]. That study suggested that *Panax ginseng (P. ginseng)* could inhibit the metabolism of DDAs in vivo. Our group has developed a new method to simultaneously detect the concentration of six aconitum alkaloids in rat plasma in 5 min. The calibration curves of each component range from 0.1 to 10 ng/mL. The method has reliable performance on the selectivity, linearity, accuracy, precision, stability, extraction recovery, and matrix effects [27], which conform to the guidelines of the National Medical Products Administration for bioanalytical assay validation. Therefore, the method could be applied to pharmacokinetic study.

In our previous work, we suggested that calcium disorder and calcium transient were observed in myocardial cells induced by AC at high doses (1–10 μmol/L), while the low doses (5–20 nmol/L) of AC could improve mitochondrial energy metabolism. These data all indicate that *Fu Zi* has the trend of high-dose toxicity and low-dose effect. AC, HA, and MA can be hydrolyzed to BAC, BHA, and BMA. In Figure 3**,** two peaks of BAC, BHA, and BMA were observed in the chromatograms. After a literature review and comparison, the phenomenon of two peaks may be caused by the hydrolysis of AC, HA, and MA by the action of the cytochrome P450 enzyme. In our previous study, we systematically studied the cytochrome P450 (CYP 450) enzyme subtypes of metabolizing AC, HA, and MA. CYP3A4 was found to be the main subtype for alkaloid (AC, HA, and MA) metabolism in vivo, and the activity of CYP3A4 is affected by the combined action of multiple alkaloids [28]. Hence, the number of various alkaloids in the body at different doses is the key to their toxicity effects.

Toxic cardiovascular effects, including arrhythmia, lower heart rate, and blood pressure, were observed after we orally administered 0.4 mg/kg of AC in rats, and these effects were not found in the low-dose (0.1 mg/kg) group [29]. The patients who experienced acute poisoning by *Fu Zi* ingestion presented with excessive salivation, arrhythmia, premature ventricular contraction, and shallow breathing [30,31]. In the pharmacodynamics pre-experiments, we found that a high dose of *Fu Zi* (5 g/kg/d) had obvious toxicity to normal mice but could partially restore the heart function of myocardial ischemia mice and improve the myocardial fibrosis of mice. Large doses of *Fu Zi* significantly enhanced the formation of autophagosomes in normal mice, but they partially inhibited the formation of autophagosomes and promoted the fusion of autophagosomes and lysosomes after the occurrence of myocardial ischemia. Compared with the poisoning symptoms of salivation and arrhythmia in the high-dose group, the rats in the low- and middle-dose groups acted normally in this study, which was similar to the clinical symptoms of *Fu Zi* high dose with toxicity while low dose with treatment. Six alkaloids in the low-dose group were eliminated within 48 h, while there was still limited residue in the high-dose group at 48 h. It was found that the pharmacokinetic parameters of three MDAs were similar [32], with small C_max,_ T_max_, and greater t_1/2_ indicating that the three MDAs can be absorbed rapidly and are difficult to be metabolized or excreted. However, the low C_max_ indicated that the bioavailability of the MDAs will be very low, and their absorption may be inhibited by some transport proteins. By incubating three MDAs in rat liver microsome, which cannot be metabolized in vivo. The CYP3A4 enzyme unit may be the key element for metabolizing the MDAs. Researchers also found that P-gp inhibits the absorption of MDAs by using a Caco-2 transwell model. The pharmacokinetic characteristics of different doses of *Fu Zi* extracts in SD rats may be related to the binding mechanism of the main components of *Fu Zi* and transporter in vivo.

It was found that intestinal metabolic profiles of the DDAs varied more than the MDAs. No glucuronide metabolites were detected, which agreed with the human liver microsome (HLM) metabolism [33]. Similarly, we observed bimodal phenomena in the plasma concentration–time curves of the six aconitum alkaloids, which was consistent with previous reports.

LQTS mutations can cause syncope and sudden death by prolonging the cardiac action potential (AP). Researchers found that myocytes carrying an LQT2 mutation (HERG-A422T), APS, and [Ca^2+^] i transients were prolonged in parallel, compared with the normal groups. LQTS models are difficult to replicate in drug evaluation studies [34], but hiPSCs make it possible to rapidly detect the toxicity or efficiency of compounds in vitro for LQTS patients. Rather than using drug-induced LQTS rat models, human LQTS cardiomyocytes come from clinical patients, which are completely consistent with the pathogenesis of patients. The importance of applying human cardiomyocyte models for evaluating the effects of various drugs has been receiving unprecedented attention. A variety of research on the mechanism of arrhythmia in human cardiomyocytes compared to that from other animals, particularly rodents, reveals the intra-species variations in cardiac electrophysiology.

Early studies have shown that AC can improve arrhythmia in patients by affecting calcium channels, but whether AC will accelerate the deterioration of LQTS mutations has not yet been reported. In our preliminary experiment, several dose groups were set up with AC concentrations ranging from 0.05–0.5 μM, which would not cause death but the doses of AC are cytotoxic. The present work suggested that 0.05–0.5 μM concentrations did not cause significant myocardial cell death during the 48 h observation period. The concentration of 0.05 μM had a significant effect on the cells, which showed higher contraction frequency and lower contraction force than the control group. The cell frequency and contractility were significantly increased at the 0.5 μM concentration. The cell contractility decreased to zero using the 5 μM concentration, and it was very weak within 30 h after treatment of AC (Figure 5). This does not imply that AC is more toxic on LQTS than normal because the LQTS cardiomyocytes also lose a substantial amount of contractility over time. The beat rate is accelerated earlier due to the effect of AC (0.5 μM) on patients with LQTS cardiomyocytes, as compared with normal hiPSC-CMs; however, it is still not clear why the cell index did not change even though the amplitude and beating rate were reduced by AC (5 μM). Compared with the normal control, the AC had no significant effect on the amplitude and frequency of calcium transient in normal hiPSC-CMs, but it could significantly increase the frequency of calcium transient in cardiomyocytes of LQTS at 5 μM (Figure 8). This explains why the fluctuation anomaly appears earlier in LQTS cardiomyocytes as compared with AC in normal hiPSC-CMs.

We further detected the effect of AC on L-type calcium current in both normal hiPSC-CMs and LQTS hiPSC-CMs, and it showed similar blockage, which suggested that L-type calcium current may not be the major target on the effect of AC on LQTS. This study has some limitations. First, we did not examine diverse types of LQTS mutations. Second, Na^+^ channels and K^+^ channels were not involved in the experiment. Therefore, we encourage additional studies examining diverse types of LQTS mutations, and these studies should supplement the data of other ion channels. In addition, although KCNQ1, KCNH2 (K^+^ channels), and the SCN5A (Na^+^ channel) also play important roles in LQTS, we only observed the effect of AC on the L-type Ca channel alone in LQTS hiPSC-CMs; therefore, K^+^ channels, such as KCNQ1 and KCNH2, and Na^+^ channels, such as SCN5A, must be observed in further studies.

In summary, there are still some deficiencies in our study despite the use of eight rats in each group. The plasma concentrations at some points on the drug concentration–time curves showed a large deviation, which may be due to individual differences in the drug resistance of the rats or an insufficient sample size. Nevertheless, research on this type of disease model is more practical and meaningful, so we will conduct a pharmacokinetic study of *Fu Zi* extracts in rats with heart failure in the future. Moreover, the effects of *Fu Zi* in healthy and LQTS cardiomyocytes may be caused by various alkaloids, such as HA and MA, so we will examine the results of other alkaloids in our upcoming work. The results of the present study show the parameters of six aconitum alkaloids in *Fu Zi* at multiple doses for the first time, which could provide support for the safe clinical application of *Fu Zi*. Moreover, for the first time we compared the effects of AC in normal and LQT-hiPSC-CMs, which may provide data for the basic research of drugs containing AC.

## 4. Materials and Methods

### 4.1. Materials and Instruments

The standard compounds (purity ≥ 98%) of AC (MUST-17110910), HA (MUST-20052710), MA (MUST-19080210), BAC (MUST-19103010), BHA (MUST-200331 11), and BMA (MUST-20022710) were purchased from Chengdu Must Bio-Technology Co., Ltd. (Chengdu, China). *Fu Zi* was obtained from Tongrentang (Beijing, China), which was identified by Professor Ma Bai ping from Beijing Institution of Radiation. Acetonitrile, methanol and formic acid of HPLC grade were purchased from Fisher Scientific Company Inc. (Boston, MA, USA). Ultrapure water was produced by a Milli-Q Reagent Water System (Millipore, Bedford, MA, USA).

The UPLC–MS/MS system consisted of a Waters ACQUITY I-Class UPLC system connected to a Waters (Milford, MA, USA) Xevo TQ-S triple quadrupole mass spectrometer with a degasser, a binary pump, flow-through-needle sample manager, a column oven with temperature control, and a cooling autosampler.

### 4.2. Preparation of Fu Zi Extracts

*Fu Zi* (200 g) was taken and broken by a pulverizer to the size of 0.5–1.0 cm. The broken *Fu Zi* was soaked in 1.6 L distilled water and boiled for 0.5 h. The filtrate was collected and the previous step was repeated for the filtered residue. The mixed filtrates were precipitated with 60% ethanol, and then centrifuged (2000× *g*, 10 min) [22]. The supernatant was decompressed and concentrated to a final density of 2.0 g/mL by the amount of the crude drugs. The final concentration of ethanol in *Fu Zi* extracts before administration was below 0.5%. The extract was subsequently kept at −20 °C for oral administration to rats. The contents of AC, HA, MA, BAC, BHA, BMA in *Fu Zi* extracts were detected to be 0.140, 0.475, 0.525, 0.910, 0.234, and 0.571 mg/g by UPLC-MS/MS, respectively.

### 4.3. Conditions of Chromatographic and Mass Spectrometry

All samples were analyzed on UPLC^®^ I-Class and Xevo^®^ TQS mass spectrometry combined system. Separations by a Waters ACQUITY UPLC^®^ HSS T3(2.1 mm × 100 mm, 1.8 μm) column were conducted with a mobile phase of water (A) and acetonitrile (B), both containing 0.1% formic acid, at the flow rate of 0.3 mL·min^−1^.The run time was 5.0 min and the gradient elution program was adopted as follows: 0–0.3 min, 10% B; 0.3–1 min, 10–50% B; 1–2.5 min, 50–90% B; 2.5–3 min, 90% B; 3–5 min, 10% B. The temperature of the column and samples were set at 45 °C and 10 °C, respectively. The volume of injection was 2 μL.

The detection was quantified by a triple quadrupole mass spectrometer that was set as multiple reactive monitoring (MRM) in the electrospray positive ionization mode (ESI+). The correlative parameters were as follows: capillary voltage: 3 kV; desolvation gas flow: 800 L·h^−1^; cone gas flow: 150 L·h^−1^; source temperature: 150 °C; desolvation temperature: 500 °C. Quantitative parameters are listed in Table 6.

### 4.4. Preparation of Calibration Standards and Quality Control Samples

The stock solutions of the six alkaloids were prepared in methanol at the concentration of 2.0 mg/mL individually. Then working and quality control solutions were obtained by serial dilution of the stock solutions with 50% acetonitrile. All solutions were stored at −20 °C.

The standard curves of the six alkaloids were in the linear range from 0.1 to 10 ng/mL (0.1, 0.25, 0.5, 1.0, 2.5, 5.0, and 10 ng/mL). The QC samples were prepared by adding the corresponding level of QC working solutions to the blank plasma, consisting of low-QC (0.2 ng/mL), mid-QC (2 ng/mL), and high-QC (8 ng/mL) samples. To obtain the plasma matrix samples with a serial of concentrations, 10 μL of a 10-fold concentration of the calibrators was added to 90 μL of blank plasma from SD rats. Standards curves and QC samples were prepared for every validation assay and sample test.

### 4.5. Pre-Treatment of Plasma Samples

The method of liquid–liquid extraction was utilized [23]. An amount of 100 μL of plasma samples from rats after oral administration of *Fu Zi* extracts was precipitated with 300 μL of 50% acetonitrile plus 50% methanol, vortexed for 1.0 min and centrifuged at a speed of 12,000× *g* at 4 °C for 10 min. The supernatant was transferred to another 1.5 mL centrifuge tube, dried with a stream of nitrogen at 40 °C, redissolved in 50 μL methanol and centrifuged at 12,000× *g* for 10 min. Then, the supernatant was pipetted out into the autosampler vials and analyzed by UPLC-MS/MS system.

### 4.6. Method Validation

To ensure that there were no endogenous compounds, the selectivity of the six alkaloids was determined by comparing the chromatograms of blank plasma samples, spiked plasma samples, and plasma samples after oral administration of *Fu Zi* extracts.

The calibration curves at a range from 0.1 to 10 ng/mL were evaluated by least-square regression using 1/x^2^ as a weighting factor. The quality control samples, including low-QC (0.2 ng/mL), mid-QC (2 ng/mL), and high-QC (8 ng/mL) samples, were prepared from another stock solution independently. The LLOQ signal-to-noise ratio was above 10 and the deviation did not exceed 20%.

The accuracy and precision were determined by LLOQ and QC samples at high, middle, and low concentrations (each for six replicate analyses) in intra-day and inter-day batches. The inter-day accuracy and precision should be assessed by three batches for at least 2 days. The accuracy [RE (%)] and precision [RSD (%)] for QC samples were required within ±15%, while for LLOQ samples, they were less than 20%.

The stability was assessed by analyzing the QC samples at high and low concentrations under different storage conditions: at room temperature for 4 h, in the autosampler after preparation for 10 h, after three freeze-thaw cycles and at −20 °C for 1 week, respectively. The RE (%) comparing the standard curve on the detection day was required to be within ± 15% in the stability test.

The matrix effect was calculated by comparing the difference of response between samples of 50% methanol and the supernatant of precipitated blank plasma with the addition of high, middle, and low QC samples, respectively. The extraction recovery was assessed by comparing the difference of peak areas between samples of 50% methanol and blank plasma with the addition of QC samples at high, middle, and low QC samples. Both RSD (%) of each QC sample should be less than 15%.

### 4.7. Animals and Treatments

24 Sprague Dawley rats (half male and female, weight 180–220 g) were purchased from Beijing Weitong Lihua Biotechnology Co.Ltd. (Beijing, China). The license number of experimental animal production was SCXK (Jing) 2016–0006. The animals were maintained under a standard laboratory condition (humidity: 40–70%, room temperature: 20–26 °C, relative humidity: 45–65%, 12 h dark-light cycle) and fed separately. All animal experiments were conducted according to the Guide for the Care and Use of Laboratory Animals (National Research Council of the USA, 1996) and approved by the Institutional Animal Care and Use Committee (IACUC-DWZX-2020-776). After 5 days of acclimation, the rats were fasted for 12 h but provided with water freely before the experiments. The rats were randomly divided into 3 groups: high (2.0 g/kg), middle (1.0 g/kg), and low (0.5 g/kg) dose groups. The *Fu Zi* extracts was administered to each rat intragastrically. An equivalent of 0.20 mL blood samples were collected from the fossa orbitalis vein into 1.5 mL heparinized centrifuge tubes at the designated time points after dosing: 0.08, 0.25, 0.5, 1, 2, 4, 8, 12, 24, and 48 h. The plasma samples were pipetted out into another 1.5 mL centrifuge tubes after being centrifuged at 5000× *g* for 10 min and then stored at −20 °C until further analysis.

### 4.8. Culture and Maintenance of Undifferentiated hiPSCs

HiPSCs derived from healthy controls (Cat: NC20010002; Lot.HJ210577NC22), or patients (Cat. NC20300002; Lot. CP210578NC04) diagnosed with LQT syndrome was maintained in HELP4004 (HELP, Nanjing, China) medium on Matrigel-coated (BD Bio science, San Jose, CA, USA) plates at 37 °C; with 5% CO_2_ (*v*/*v*).

### 4.9. Impedance Beating Recordings

The cardiomyocytes were seeded on Nanion CardioExcyte 96 Sensor Plates (NSP-96, Nanion Technologies, Munich, Germany) after 0.1% gelatin coating for 1 h. The initial volume was 200 μL per well and contained approximately 100 million cells. After seeding, the NSP-96 and CardioExcyte 96 were returned to the incubator. All cells were replaced with fresh medium 200 μL per well every 48 h and tested after 168 h. Cellular index, beat rate, base impedance and beat amplitude were monitored in this experiment.

### 4.10. Ca^2+^ Imaging

Dissociated day 30–33 post-differentiation hiPSC-CMs (healthy and LQT specific patient) were reseeded Matrigel-coated plate in HELP4004 (HELP) and were treated with 5 μM Fluo-3 AM and 0.02% Pluronic (Life Technologies) in DMEM/F12 (Gibco) for 60 min at 37 °C. Cells were washed with DMEM/F12 without fluorochrome solution afterwards for 30 min, replaced the solution with Tyrode’s solution. Ca^2+^ imaging was conducted using Olympus IX71 confocal microscope (Olympus IX71, Tokyo, Japan) and analyzed using Olympus imaging software. Ca^2+^ spontaneous transients were obtained using a single cell line scan mode at 37 °C.

### 4.11. Patch-Clamp Recordings of hiPSCs

Standard patch-clamp techniques were used to record action potentials, and L-type calcium currents. Composition of intracellular fluid and extracellular fluid were shown in Table 7 and Table 8, respectively. The signal was amplified using an HEKA EPC-10 patch-clamp amplifier (HEKA, Electronics, Ludwigshafen, Germany) and low-pass filtered at 5 kHz. Patch pipettes were fabricated from glass capillaries using a Sutter P-97 microelectrode puller (Novato, CA, USA) and the tips were heat polished with a microforge (NARISHIGE MF-900) to gain a resistance of 2–5 MΩ. Data acquisition was achieved using HEKA Patchmaster (V2x73.2). Data analysis and fit were performed using Clamfit and Graphpad Prism 6.0. The experiment was conducted at 37 °C. One cell can test one or more drugs, or multiple concentrations of the same drug but needs to be rinsed with extracellular fluid between each test. Nifedipine (Sigma) was used as a positive control to ensure cell quality. External solution containing 0.1% DMSO was applied as vehicle to establish the baseline. Recording was done at a temperature-controlled room of 35–37 degrees Celsius.

### 4.12. Compound Solutions in Drug Assay

Isoproterenol, nifedipine, and AC were purchased from Sigma-Aldrich, which were resolved in DMSO (Sigma-Aldrich, D2650). Drug compounds final concentration were AC (0.05, 0.5,5 μM), nifedipine (1 μM), isoproterenol (3 μM), respectively. Dilutions were prepared within 60 min of treatment. All the compound solutions were subjected to regular 10 min ultrasound and oscillations to ensure that compounds were completely dissolved. Equal amounts of DMSO (0.1%) were used as the vehicle controls.

### 4.13. Statistical Analysis

The data were acquired by Mass Hunter Workstation Software from Waters (UNIFI). Microsoft Office Excel and the SPSS software (Version 13.0) was used for data analysis. The data below the LLOQ were regarded as zero in summary statistics. The pharmacokinetic parameters were calculated by DAS pharmacokinetic program (Version 2.0) and all analyzed by non-compartmental method. The figures were displayed and processed by GraphPad prisms ware (Version 5.01). All data were presented as mean ± SD. One-way analysis of variance was used to detect significant differences between the high, middle, and low dose groups. Statistical analysis was determined by paired or unpaired Student’s *t*-test (two tail) for comparison between two groups. Unless otherwise specified, statistically significant differences defined by * *p* < 0.05 or ** *p* < 0.01 or *** *p* < 0.001.

## 5. Conclusions

A rapid, sensitive, and reproducible method to simultaneously detect the concentration of AC, HA, MA, BAC, BHA, and BMA in biological samples has been developed, validated, and then employed successfully. The pharmacokinetic characteristics of six aconitum alkaloids after oral administration of *Fu Zi* extracts in SD rats have shown significant differences between the high- and low-dose group. AC had no significant effect on the amplitude and frequency of calcium transient in normal hiPSC-CMs, but it could significantly increase the frequency of calcium transient in cardiomyocytes of LQTS at 5 μM. The six aconitum alkaloids in the high group could be more quickly absorbed, more slowly eliminated, and more widely distributed. The pharmacokinetics data could provide valuable information for the safe application and clinical treatment of *Fu Zi*.

## Figures and Tables

**Table 1:** The calibration equations, linear ranges, correlation coefficients, LLOQ, and LOD of the six compounds in rat plasma.

Compounds	Calibration Equation	Correlation Coefficient (r)	Linear Range (ng/mL)	LLOQ (ng/mL)	LOD (ng/mL)
AC	y = 4.82 × 10^5^x + 1.66 × 10^4^	0.9994	0.1–10	0.1	0.025
HA	y = 7.63 × 10^5^x + 2.29 × 10^4^	0.9990	0.1–10	0.1	0.025
MA	y = 6.34 × 10^5^x + 2.46 × 10^4^	0.9984	0.1–10	0.1	0.025
BAC	y = 3.66 × 10^5^x + 1.63 × 10^4^	0.9992	0.1–10	0.1	0.025
BHA	y = 9.78 × 10^5^x + 7.3 × 10^4^	0.9985	0.1–10	0.1	0.025
BMA	y = 1.23 × 10^5^x + 5.49 × 10^5^	0.9992	0.1–10	0.1	0.025

**Table 2:** Intra- and Inter-day precision and accuracy of the developed methods (*n* = 6).

Compounds	Conc. (ng/mL)	Intra-Day			Inter-Day		
		Measured Conc. (ng/mL)	Accuracy (RE, %)	Precision (%)	Measured Conc. (ng/mL)	Accuracy (RE, %)	Precision (%)
AC	0.1	0.11 ± 0.01	9.2	6.2	0.11 ± 0.01	11.8	8.4
	0.2	0.21 ± 0.02	7.3	7.6	0.21 ± 0.02	5.3	10.5
	2	2.20 ± 0.30	10.0	13.8	2.26 ± 0.23	12.8	10.0
	8	9.00 ± 0.38	12.5	4.2	8.34 ± 0.79	4.3	9.5
HA	0.1	0.09 ± 0.01	9.8	6.3	0.11 ± 0.02	7.7	13.6
	0.2	0.21 ± 0.01	7.1	3.9	0.23 ± 0.02	13.8	9.4
	2	2.28 ± 0.06	14.1	2.5	2.19 ± 0.29	9.5	13.3
	8	8.06 ± 0.41	0.9	5.1	8.74 ± 0.62	9.3	7.1
MA	0.1	0.10 ± 0.01	−4.7	6.1	0.08 ± 0.01	−15.7	11.5
	0.2	0.20 ± 0.02	−1.2	8	0.20 ± 0.03	−0.6	12.9
	2	2.19 ± 0.06	9.3	2.8	2.17 ± 0.23	8.6	10.3
	8	7.50 ± 0.40	−6.3	5.3	7.86 ± 0.50	−1.8	6.3
BAC	0.1	0.09 ± 0.01	−7.5	7.8	0.11 ± 0.02	6.7	18.8
	0.2	0.22 ± 0.02	10.3	11.0	0.21 ± 0.03	6.9	13.3
	2	2.24 ± 0.17	11.9	7.5	2.27 ± 0.20	13.7	8.8
	8	8.53 ± 0.60	6.6	7.1	8.63 ± 0.60	7.9	6.9
BHA	0.1	0.09 ± 0.01	−8.7	4.7	0.09 ± 0.01	−9.4	9.2
	0.2	0.20 ± 0.01	−2.3	3.9	0.20 ± 0.02	3.4	8.0
	2	2.29 ± 0.05	14.7	2.2	2.17 ± 0.14	8.5	6.3
	8	8.50 ± 0.25	6.2	2.9	7.91 ± 0.58	−1.2	7.3
BMA	0.1	0.09 ± 0.01	−11.3	8.8	0.09 ± 0.01	−6.0	11.7
	0.2	0.19 ± 0.02	−0.4	9.1	0.21 ± 0.02	4.8	9.8
	2	2.07 ± 0.30	3.4	14.6	2.25 ± 0.27	12.3	12.2
	8	8.08 ± 0.40	0.19	4.9	8.52 ± 0.76	6.5	9.0

**Table 3:** Stability of the developed methods (*n* = 6).

Compounds	Conc. (ng/mL)	4 h at Room Temperature			10 h in Autosampler			7 Days of Storage at −20 °C			Freeze-Thaw Cycles		
		Measured Conc. (ng/mL)	Accuracy (RE, %)	Precision (%)	Measured Conc. (ng/mL)	Accuracy (RE, %)	Precision (%)	Measured Conc. (ng/mL)	Accuracy (RE, %)	Precision (%)	Measured Conc. (ng/mL)	Accuracy (RE, %)	Precision (%)
AC	0.2	0.21 ± 0.01	3.6	4.2	0.18 ± 0.01	−8.9	5.2	0.21 ± 0.01	5.6	6.1	0.19 ± 0.02	−5.2	9.0
	8	7.56 ± 0.30	−5.5	3.9	8.29 ± 0.24	3.7	2.9	8.23 ± 1.08	2.8	13.1	8.40 ± 0.41	4.9	4.9
HA	0.2	0.22 ± 0.01	11.2	6.2	0.19 ± 0.02	−3.0	7.7	0.20 ± 0.02	−1.0	10.6	0.19 ± 0.02	−2.8	12.6
	8	7.60 ± 0.33	−5.0	4.3	8.34 ± 0.31	4.2	3.7	7.86 ± 0.82	−1.8	10.5	8.26 ± 0.34	3.2	4.1
MA	0.2	0.19 ± 0.01	−4.2	5.2	0.18 ± 0.01	−8.7	7.4	0.19 ± 0.01	−6.7	5.8	0.18 ± 0.01	−9.6	7.2
	8	7.03 ± 0.34	−12.1	4.8	8.12 ± 0.24	1.5	2.9	7.50 ± 0.89	−6.2	11.9	8.25 ± 0.35	3.1	4.2
BAC	0.2	0.19 ± 0.01	−3.0	6.2	0.19 ± 0.02	−3.1	12.4	0.18 ± 0.01	−10.5	5.5	0.18 ± 0.01	−10.8	6.8
	8	7.02 ± 0.37	−12.2	5.2	8.29 ± 0.26	3.6	3.1	7.38 ± 0.91	−7.8	12.4	8.31 ± 0.37	3.9	4.5
BHA	0.2	0.21 ± 0.02	4.3	10.7	0.20 ± 0.01	−2.3	3.9	0.19 ± 0.01	−4.9	3.0	0.19 ± 0.01	−1.8	6.8
	8	7.67 ± 0.76	−4.1	9.9	8.50 ± 0.25	6.2	2.9	8.01 ± 0.23	0.1	2.9	7.69 ± 0.38	6.7	3.8
BMA	0.2	0.22 ± 0.02	11.4	10.7	0.18 ± 0.01	−10.4	7.9	0.19 ± 0.01	−3.1	6.5	0.20 ± 0.02	−2.5	10.6
	8	8.12 ± 0.69	1.5	8.5	7.79 ± 0.31	−2.6	4.0	7.40 ± 0.75	−7.4	10.2	7.69 ± 0.38	−3.9	4.9

**Table 4:** Extraction recovery and matrix effect of the developed methods (*n* = 6).

Compounds	Conc. (ng/mL)	Extraction Recovery (%)	Precision (%)	Matrix Effect (%)	Precision (%)
AC	0.2	75.1 ± 5.9	7.8	85.2 ± 8.3	9.8
	2	88.2 ± 7.0	8.0	91.3 ± 8.9	9.8
	8	90.1 ± 4.1	4.5	87.8 ± 7.4	8.5
HA	0.2	78.5 ± 7.3	9.4	81.5 ± 11.4	14.0
	2	85.3 ± 4.2	5.0	84.9 ± 9.0	10.6
	8	89.7 ± 4.4	4.9	86.9 ± 9.4	10.8
MA	0.2	73.8 ± 6.7	9.1	75.6 ± 8.7	11.5
	2	79.7 ± 5.1	6.4	79.0 ± 5.5	6.9
	8	79.7 ± 3.8	4.7	79.5 ± 7	8.8
BAC	0.2	88.3 ± 6.8	7.7	93.2 ± 10.5	11.3
	2	89.1 ± 5.8	6.5	90.2 ± 7.2	8.0
	8	90.0 ± 4.4	4.9	89.0 ± 6.8	7.6
BHA	0.2	67.7 ± 4.0	5.8	67.6 ± 8.8	13.0
	2	95.0 ± 7.2	7.6	100.3 ± 6.9	6.9
	8	101.1 ± 5.5	5.4	97.3 ± 6.0	6.1
BMA	0.2	82.0 ± 5.7	7.0	85.7 ± 8.5	9.9
	2	88.8 ± 6.6	7.5	89.3 ± 5.7	6.4
	8	88.7 ± 5.2	5.9	85.5 ± 5.7	6.7

**Table 5:** The main pharmacokinetic parameters of the six compounds in SD rat plasma at different oral doses of 0.5 g/kg, 1.0 g/kg and 2.0 g/kg of *Fu Zi* extracts (mean ± SD, *n* = 8).

Compounds	Dose (g/kg)	C_max_ (μg/L)	T_max_ (h)	V_z_/F (L/kg)	t_1/2z_ (h)	CL_z_/F (L/h/kg)	AUC_(0–48h)_ (μg/L·h)
AC	0.5	1.28 ± 0.34	4.38 ± 3.11	14.01 ± 4.33	1.90 ± 0.31	4.62 ± 1.29	13.47 ± 3.28
	1.0	1.69 ± 0.33	3.13 ± 1.25	70.03 ± 33.39 *	7.53 ± 2.74 *	6.39 ± 1.83	17.15 ± 4.68
	2.0	2.27 ± 0.70 *	1.03 ± 0.84 ^##^	166.81 ± 80.09 **^,#^	11.47 ± 4.30 *	8.85 ± 2.11 **	23.82 ± 7.77 *
HA	0.5	6.37 ± 1.96	2.14 ± 0.90	12.75 ± 6.88	1.83 ± 0.64	4.02 ± 1.80	62.90 ± 26.89
	1.0	9.22 ± 1.78 *	3.88 ± 2.03	45.31 ± 26.28 *	8.72 ± 3.40 **	3.57 ± 1.05	115.20 ± 24.99 **
	2.0	12.48 ± 4.42 *	1.60 ± 1.47	80.41 ± 35.49 **	11.92 ± 4.59 **	4.78 ± 1.73	185.72 ± 64.72 **
MA	0.5	2.66 ± 0.50	2.18 ± 1.40	33.10 ± 25.06	1.72 ± 0.25	9.89 ± 1.93	26.39 ± 5.42
	1.0	4.41 ± 1.81	3.86 ± 2.19	52.99 ± 26.92	4.27 ± 2.27	8.73 ± 1.00	59.41 ± 9.25 **
	2.0	6.77 ± 1.32 **^,#^	1.06 ± 0.81 ^#^	87.22 ± 43.08 *	6.04 ± 3.85 *	12.49 ± 3.75	85.82 ± 20.71 **^,#^
BAC	0.5	1.52 ± 0.71	3.72 ± 3.60	193.64 ± 116.81	4.48 ± 0.64	27.59 ± 14.27	16.21 ± 5.43
	1.0	3.24 ± 2.01	5.71 ± 5.07	151.61 ± 97.07	5.91 ± 3.79	16.24 ± 7.33	38.51 ± 15.45 *
	2.0	5.91 ± 2.25 **	0.34 ± 0.20	800.57 ± 396.01 *^,#^	13.67 ± 4.26 **^,#^	34.98 ± 17.89	50.54 ± 19.45 **
BHA	0.5	BQL	BQL	BQL	BQL	BQL	BQL
	1.0	0.41 ± 0.13	4.10 ± 3.88	257.62 ± 106.42	7.70 ± 4.28	30.60 ± 17.16	7.96 ± 3.58
	2.0	1.52 ± 0.76 ^#^	0.40 ± 0.17	383.54 ± 177.00	8.75 ± 7.14	25.58 ± 18.42	17.29 ± 6.18 ^#^
BMA	0.5	0.78 ± 0.26	5.18 ± 4.84	207.34 ± 75.96	4.53 ± 3.84	44.39 ± 16.02	6.85 ± 2.40
	1.0	1.92 ± 0.92 *	6.50 ± 5.93	382.12 ± 166.25	16.81 ± 10.13 *	19.81 ± 13.56 *	28.16 ± 13.38 *
	2.0	6.92 ± 3.00 **^,##^	0.35 ± 0.17	616.61 ± 290.20 *	19.14 ± 8.84 **	26.10 ± 13.73	44.20 ± 18.82 *

* *p* < 0.05, ** *p* < 0.01 versus 0.5 g/kg; ^#^ *p* < 0.05, ^##^ *p* < 0.01 versus 1.0 g/kg. BQL: Below lower limit of quantitation.

**Table 6:** Mass Spectra Properties of the Compounds.

Compounds	Q1/Q3 (m/z)	Cone(v)	C.E. (V)
AC	646.24/104.90	58	69
HA	616.24/104.85	58	46
MA	632.23/104.81	58	33
BAC	604.32/104.81	52	43
BHA	574.24/104.85	48	52
BMA	590.30/104.81	50	20

**Table 7:** Composition of intracellular fluid and extracellular fluid (L-type calcium channels).

Reagent	Extracellular Solution (mM) (EC 0.0.0 NaCl-Ringer’s Solution)	Intracellular Solution (mM) (IC 0.0.0 KCl-Ringer’s Solution)
CsCl	20	135
TEA-Cl	140	2
BaCl2	5	-
glucose	25	-
HEPES	10	10
CaCl2	-	2
Mg2ATP	-	3
NaCl	-	10
EGTA	-	5
pH	7.3 (Adjust with CSOH)	7.2 (Adjust with CSOH)
Osmotic pressure	~315 mOsm	~300 mOsm

**Table 8:** Composition of intracellular fluid and extracellular fluid (action potential).

Reagent	Extracellular (mM) (EC 0.0.0 NaCl-Ringer’s Solution)	Intracellular (mM) (IC 0.0.0 KCl-Ringer’s Solution)
NaCl	140	-
KCl	5	20
MgCl2.6H2O	1	1
HEPES	5	10
glucose	10	-
CaCl2	1.8	-
K-Asparate	-	110
Na2-phosphocreatine	-	4
EGTA	-	5
Na-GTP	-	0.1
Mg2-ATP	-	5
pH	7.3 (Adjust with CSOH)	7.3(Adjust with CSOH)
Osmotic pressure	~315 mOsm	~300 mOsm

## Data Availability

The data used to support the findings of this study are available from the corresponding author upon request.

## Abbreviations

Aconiti Lateralis Radix Praeparata	*Fu Zi*
Aconitine	AC
Acquired Long QT Syndrome	aLQTS
AP duration	APD
Benzoylaconine	BAC
Benzoylhypaconine	BHA
Benzoylmesaconine	BMA
Calcium-induced calcium release	CICR
Concentration	Conc.
Congenital long QT syndrome	cLQTS
Cytochrome P450	CYP450
Diester-diterpene alkaloids	DDAs
Monoester-diterpene alkaloids	MDAs
Hypaconitine	HA
Half life time	t1/2
Human induced pluripotent stem cells	hiPSCs
Long QT syndrome	LQTS
Lower limit of quantitation	LLOQ
Limit of detection	LOD
Maximum concentration	Cmax
Mesaconitine	MA
Multiple reaction monitoring	MRM
*Panax ginseng*	*P. ginseng*
Peak time	Tmax
Relative standard deviation	RSD
Relative error	RE
The area under the curve	AUC
Traditional Chinese Medicine	TCM
Ultra performance liquid chromatography tandem mass spectrometry	UPLC-MS/MS
Volume of distribution	Vz/F
